# Using UPLC-MS/MS for Characterization of Active Components in Extracts of Yupingfeng and Application to a Comparative Pharmacokinetic Study in Rat Plasma after Oral Administration

**DOI:** 10.3390/molecules22050810

**Published:** 2017-05-17

**Authors:** Meng-Qi Jia, Ye-Juan Xiong, Yun Xue, Yan Wang, Chao Yan

**Affiliations:** 1 School of Pharmacy, Shanghai Jiao Tong University, Shanghai 200240, China; forlorn77@sjtu.edu.cn (M.-Q.J.); my19870627@sjtu.edu.cn (Y.X.); chaoyan@sjtu.edu.cn (C.Y.); 2 Shanghai University of Medicine & Health Sciences, Shanghai 201318, China; daisyxyj@163.com

**Keywords:** Yupingfeng formula, chemical constituents, liquid chromatography, tandem mass spectrometry, integrated pharmacokinetics

## Abstract

Yupingfeng (YPF), a famous traditional Chinese medicine, which contains a large array of compounds, has been effectually used in health protection. A two-dimensional liquid chromatography (^2^D-LC) combined with quadrupole time-of-flight mass spectrometry (QTOF-MS) method was firstly established to separate and identify chemical components in YPF. A total of 33 compounds were identified, including 15 constituents (flavonoids and saponins) in *Astragali radix*; seven constituents (sesquiterpenoids and polysaccharide) in *Atractylodis rhizoma*; and 11 constituents (chromone and coumarins) in *Saposhnikoviae radix*. The corresponding fragmentation pathway of typical substances was investigated. Then, seven active constituents (astragaloside, calycosin, formononetin, cimicifugoside, 4-*O*-beta-d-glucosyl-5-*O*-methylvisamminol, sec-*O*-glucosylhamaudol, and atractylenolide II) derived from three medicinal plants were chosen to further investigate the pharmacokinetic behavior of YPF formula using ultrahigh-performance liquid chromatography with triple quadrupole mass spectrometry system. The method was sensitive, accurate and reliable. We also used the area under the plasma concentration–time curve from zero to infinity (AUC_0−∞_) as weighting factor to make an integrated pharmacokinetic curve. Results show that the constituents of *Saposhnikoviae radix* have the best absorption and pharmacokinetic behavior and may play important role in leading to the changes of overall therapeutic effects of YPF. Further study is needed to confirm the association between them.

## 1. Introduction

Currently, traditional Chinese medicine (TCM) has drawn more and more attention worldwide. TCM is usually prepared as a formula by unique methods with a specific combination which contains multiple herbs [1,2,3,4]. The multiple components in a formula work together to achieve satisfactory therapeutic efficacy and minimize adverse reactions [5]. However, their synergistic mechanisms are poorly understood and must be addressed using a more holistic approach [6]. Pharmacokinetic studies might further provide physiologically relevant clues to the target identification and mechanistic study of TCM formula [7].

Yupingfeng (YPF), a famous TCM formula, has been efficaciously used in health protection, immunity enhancement and inflammatory disease control for thousands of years [8,9,10], including repeated relapses of respiratory infection, allergic rhinitis, chronic bronchitis and so on. It also plays an important role when severe acute respiratory syndrome (SARS) and avian influenza (H7N9) epidemic outbreak in China. It contains three unique medicinal plants (Figure S1), *Astragali radix*, *Atractylodis rhizoma* and *Saposhnikoviae radix*, in a ratio of 3:1:1 by weight of dried plants. Furthermore, numerous pharmacological studies exhibited that there were varieties of flavonoids and saponins in *Astragali radix* [11,12,13,14,15]; sesquiterpenoids in *Atractylodis rhizoma*; and chromones and coumarins [16] in *Saposhnikoviae radix*, which show anti-inflammatory, antiviral and immunosuppressive activities, as well as their glycoside counterparts [17,18,19,20]. We may assume that these kinds of constituents could explain the effectiveness of YPF formula.

Some analytical techniques based on LC-MS have been reported for the detection of several high-abundance components and their counterparts in YPF in biological samples [21,22]. Most of these reports are focused on one kind of compound in single herbal plant of YPF, especially *Astragali radix* and *Saposhnikoviae radix*, using the most abundant compounds such as saponins and their aglycones as chemical markers [23]. Only a small number of reports are available on the simultaneous analysis of the bioactive constituents in *Atractylodis rhizoma*, such as atractylenolide I–III [24,25,26], as its low content and similar retention behaviors make the detection difficult. To our knowledge, there is no report on the integrated pharmacokinetics of both these ingredients (including atractylenolide II) in three herbs has been published.

Due to its high peak capacity and accurate mass measurement, two-dimensional liquid chromatography (^2^D-LC) combine with quadrupole time-of-flight mass spectrometry (QTOF-MS) is a powerful technique, particularly in separation of complicated herbal extracts. It also can be used to identify unknown compounds. In this study, ^2^D-LC-QTOF-MS was established to separate and identify chemical compounds in YPF, and 33 compounds were identified or tentatively characterized. The corresponding fragmentation pathway of typical substances was investigated. Then, a rapid, simple and validated method using ultrahigh-performance liquid chromatography with triple quadrupole mass spectrometry (UPLC-QqQ-MS) system was developed for simultaneously quantification of astragaloside, calycosin, formononetin, atractylenolide II, cimicifugoside, 4-*O*-beta-d-glucosyl-5-*O*-methylvisamminol, and sec-*O*-glucosylhamaudol in rat plasma with relatively low concentration. We applied this method to pharmacokinetic study of YPF orally administered, and used the area under the plasma concentration–time curve from zero to infinity (AUC_0−∞_) as the weighting factor to make an integrated pharmacokinetic behavior for those main active ingredients, which was firstly elucidated.

## 2. Material and Methods

### 2.1. Drugs and Chemicals

Astragaloside, Calycosin, Formononetin, Atractylenolide II, Cimicifugoside, 4-*O*-beta-d-glucosyl-5-*O*-methylvisamminol, and Sec-*O*-glucosylhamaudol standards were purchased from National Institutes for Drug and Food Control (Shanghai, China, Series numbers: 110781-201314, 73069-13-3, 111522-201008, 111523-201208, MUST-14080402, 20575-57-9, and MUST-14030710). The purity of those reference standards were all more than 98% according to HPLC with DAD analysis. MS-grade acetonitrile, methanol, and acetic acid were purchased from Merck (Darmstadt, Germany). Deionized water was prepared by a Milli-Q system (Millipore Corporation, Billerica, MA, USA) and was used throughout. All other chemicals were of analytical grade, unless otherwise noted.

*Astragali radix* (root of *Astragalus membranaceus* the (Fisch.) Bge) Hsiao (Huangqi, 14021203), *Atractylodis rhizoma* (rhizome of *Atractylodis macrocephalae* Koidz (Baizhu, 14030303)) and *Saposhnikoviae radix* (root of *Saposhnikovia divaricate* (Turez.) Schischk (Fangfeng, 13072510)), all the dried herb were purchased from Shanghai Ley′s Pharmaceutical CO., Ltd. (Shanghai, China).

### 2.2. Preparation of YPF Extracts

The herbs were processed as previously described [23]: dried plants of *Astragali radix*, *Atractylodis rhizoma* and *Saposhnikoviae radix* (30:10:10 g) by weight were mixed and double extracted by refluxing with boiling water (1:6 and then 1:4, *w*/*v*) for 40 min; thereafter the water decoction was concentrated to 0.5 g/mL (crude drugs); and then the decoction was centrifuged for 10 min at 10,000 rpm (4 °C) after 30-min standing. The supernatant was centrifuged for 10 min at 10,000 rpm (4 °C), filtrated by the microvoid filter film (0.22 μm) before directly injected into the LC system for analysis.

### 2.3. Preparation of Standards

The primary standard stock solutions of astragaloside (1.68 mg/mL), calycosin (1.18 mg/mL), formononetin (2.00 mg/mL), atractylenolide II (1.15 mg/mL), cimicifugoside (1.25 mg/mL), 4-*O*-beta-d-glucosyl-5-*O*-methylvisamminol (1.37 mg/mL), and sec-*O*-glucosylhamaudol (1.51 mg/mL) were prepared by dissolving in methanol. These stock solutions were further appropriately diluted to give a serial of working standard solutions by spiking aliquots of the stock solutions into methanol. Quality control (QC) samples (with low, medium, and high concentration, respectively) were independently prepared. All solutions were stored at −20 °C and brought to room temperature before use.

### 2.4. Plasma Sample Preparation

For calibration standards, to an aliquot of 90 μL blank plasma sample, 10 μL of above working solutions were added to obtain final concentrations of 0.15, 0.25, 1.0, 1.5, 3.5, 6.0, 15 and 150 ng/mL of astragaloside, calycosin and cimicifugoside; 0.05, 0.1, 0.5, 1.5, 5.0, 8.0 and 80.0 ng/mL for atractylenolide II, 4-*O*-beta-d-glucosyl-5-*O*-methylvisamminol and sec-*O*-glucosylhamaudol; and 1.0, 1.5, 2.0, 5.0, 15, 20, 80 ng/mL of formononetin. Then, 300 μL acetonitrile for protein precipitation were added. For the incurred samples, 10 µL of methanol (volume of the corresponding standard solution for calibration curve) and 90 μL of samples with 300 μL acetonitrile were mixed, the mixture was then vortex-extracted for 2 min, and centrifuged for 10 min at 15,000 rpm (4 °C). The supernatant was transferred to a new tube and evaporated under a gentle stream of nitrogen to dryness. The residue was reconstituted with 80 μL of initial mobile phase, vortex-mixed for 1 min and centrifuged at 15,000 rpm for 5 min.

### 2.5. Instruments and Analytical Conditions

#### 2.5.1. ^2^D-NPLC/RPLC-QTOF-MS Analysis of Chemical Constituents in YPF

Two dimension-normal/reversed phase liquid chromatography combine with quadrupole time-of-flight mass spectrometry (^2^D-NPLC/RPLC-QTOF-MS) was used in this study. In the first dimension, TSK gel Amide-80 column (4.6 mm × 150 mm, 3 μm) was used. Mobile phase water–acetonitrile (A:B) was run at 0.8 mL/min, the initial solvent was 98% B and kept for 2 min, and then the gradient was 2–15 min, 98–87% B; 15–20 min, 87–64% B; 20–26 min, 64–60% B; 26–40 min, 60–55% B; 40–41 min, 55–98% B. An automatic switching valve was used to collect the effluent of the Amide column every 8 min, for five fractions totally, and each fraction was collected 10 times and dried by nitrogen. Afterward, the residues were redissolved in 100 µL methanol–water solution (1:1, *v*/*v*) and vortex-mixed for 1 min and centrifuged at 15,000 rpm for 10 min, then 10 μL supernatant was injected into the second dimension. In the second dimension, a Waters UPLC-BEH C18 column (100 mm × 2.1 mm, 1.7 μm) was used. The mobile phase were water (containing 0.05% formic acid)–methanol (A:B) with a flow rate of 0.4 mL/min. The gradient was 0–2 min, 5–20% B; 2–6.5 min, 20–35% B; 6.5–8 min, 35–60% B; 8.5–11 min, 60–100% B; 13–13.5 min, 100–5% B; 13.5–15.0 min, 5% B. The detection and identification were carried out on UltiMate3000 RSLCnano Liquid Chromatography/maXis impact UHR-QTOF-MS (Bruker Daltonics, Bremen, Germany; Thermofisher, Waltham, MA, USA), equipped with ESI+ source, the conditions were as follow: Capillary, 3.0 kV; Sampling Cone, 35.0 v; Extraction Cone, 3.0 v; Cone Gas Flow, 50.0 L/Hr; Source Temperature, 115 °C; Desolvation Gas Flow, 600.0 L/Hr; Desolvation Temperature, 350 °C; Collision Energy, 4.0; Wavelength range, 210 to 500 nm; Lock Mass, 556.2771. The mass range was set at 50–1000. All the acquisition and analysis of data were controlled by Masslynx V4.1 software (Waters Corporation, Milford, MA, USA) for peak detection and peak alignment.

#### 2.5.2. Quantitative Analysis of Seven Active Constituents of YPF in Plasma Samples by UPLC-QqQ-MS

The Chromatographic UPLC-QqQ-MS procedure of pharmacokinetics study was performed using ACQUITY UPLC (Waters, Milford, MA, USA) and SCIEX SelexION Triple Quad™ 5500 System (AB Sciex, Framingham, MA, USA) equipped with a heated electrospray ionization source (ESI) interface, separation was achieved using a Waters BEH C18 column (2.1 mm × 100 mm, 1.7 μm) which was protected by an online filter, the mobile phase was at a flow rate of 0.5 mL/min, consisted of aqueous solvent A: water–acetic acid (1000:1, *v*/*v*) and organic phase solvent B: acetonitrile. A 10-min binary gradient elution was performed for the separation, and the consecutive program was as follows: an isocratic elution of 5% solvent B to equilibrate the column, then followed by a linear gradient elution: 0–2.5 min, 5–27% B; 2.5–3.1 min, 27–36% B; 3.1–4.1 min, 36–57% B; 4.1–4.8 min, 57–84% B; 4.8–5.8 min, 84–100% B; 5.8–7 min, 100–100% B; 7–8 min, 100–5% B; and the re-equilibration time of gradient elution was 2 min. The column and auto-sampler tray temperature were maintained constantly at 45 °C and 4 °C, respectively. The injection volume was 10 μL. The electrospray ionization source was operated in positive mode. Quantitation was performed by schedule multiple reactions monitoring (sMRM). In the positive mode, the tandem mass spectrometry (MS/MS) typical instrumental conditions were: ion spray voltage 5.5 kV, ion source gas 150 psi, ion source gas 250 psi, temperature 500 °C, and curtain gas 38 psi. Data acquisition and processing were performed using Analyst Software (AB Sciex, Corp., Framingham, MA, USA).

### 2.6. Method Validation

The method was fully validated in accordance with the US-FDA document and other related guidelines with respect to specificity, linearity, precision and accuracy, recovery, matrix effect and stability.

Selectivity was assessed by comparing the chromatograms of a blank plasma sample, a blank plasma sample spiked with the working solutions and a rat plasma sample obtained from 15 min after an oral administration of the mix standard solution of seven analytes.

Linearity and sensitivity: The linearity of assay for the test in vivo compounds with a total of six calibration standards were prepared in blank rat plasma at least six concentrations, respectively, obtained by plotting the peak area (y) of each analyte versus the theoretical standard concentrations (x) and assessed by weighed least-squares linear regression using concentrations (x) as the weighting factor in SPSS-based software.

Accuracy and precision: The accuracy and precision of intra-day and inter-day determination were carried out in six replicates samples at three QC levels (low, medium and high) within the same day and on three consecutive days.

Matrix effect and recovery: The matrix effect of the biological matrix (rat plasma) and extraction recoveries were evaluated at three QC levels for analytes with six replicates, matrix effect was assessed via comparison of the peak responses of the analytes in the extracted blank rat plasma matrix to those obtained from neat standard solutions at equivalent concentrations. While extraction recoveries were calculated by comparing the peak areas of samples added before extraction with those of samples added after extraction.

Stability The short-term and long-term stability of seven analytes in rat plasma in auto-sampler vials for 24 h, at −20 °C for 30 days and after three freeze–thaw cycles was assessed, and also carried out in six replicates samples at three QC levels (low, medium and high).

### 2.7. Application to Pharmacokinetic Study in Rats

The developed UPLC-QqQ-MS method was applied to a pharmacokinetic study of seven analytes in rat plasma. Male healthy adult Sprague-Dawley rats (*n* = 6, 200 ± 20 g body weight) were supplied by Shanghai Jiao Tong University Laboratory Animal Centre (Shanghai, China). Animals were acclimatized in an environmental controlled breeding room with standard laboratory food and water for one week prior to the experiments. All the rats were fasted for 12 h, with free access to water prior to the experiments, animal welfare and experimental procedures conformed to guidance for the care and use of laboratory animals, similarly the ethical regulations of SJTU.

After oral administration of a single dose of 10 mg/kg (10 mL/kg) astragaloside, calycosin, formononetin, atractylenolide II, cimicifugoside, 4-*O*-beta-d-glucosyl-5-*O*-methylvisamminol, and sec-*O*-glucosylhamaudol mix standard solution prepared with 0.5% carboxymethyl cellulose sodium (CMC-Na), 200 μL of blood samples were planned to be collected manually using heparinized tubes at scheduled time points (0, 15, 30, and 45 min, and 1,1.5, 2, 3, 5, 7,12 and 24 h). The total blood volume collected during each session did not exceed 1.5% of the male rat total body weight. Then immediately centrifuged at 4000 rpm for 5 minutes, plasma was transferred into another via and stored at −80 °C until assay.

### 2.8. Pharmacokinetic Data Analysis

The plasma concentrations of seven analytes at different time points are described as the mean ± s.d. The concentration versus time curve was plotted. Compartmental model was chosen to calculate all the related pharmacokinetic parameters including C_max_ (ng/mL), T_max_ (h), AUC_0−∞_ (ng·h/mL), CL/F (L/h.kg), T_1/2α_ (h) and T_1/2β_ (h) were calculated by Kinetica4.4 software (Thermo Fisher Scientific™, Corp., Waltham, MA, USA), and performed with GraphPad 6 (GraphPad Software, Inc., La Jolla, CA, USA). We also use the area under the plasma concentration–time curve from zero to infinity (AUC_0−∞_) as the weighting factor to make an integrated pharmacokinetic curve. By plugging various AUC_0−∞_ values into the following equations, integrated concentration of total analytes was calculated:

*ω_j_* = AUC_j0−∞_/∑AUC_j0−∞_ (1)

∑AUC_j0−∞_ = UC_a0−∞_ + AUC_b0−∞_ + AUC_c0−∞_ + AUC_d0−∞_ + AUC_e0−∞_ + AUC_f0−∞_ + AUC_g0−∞_ (2)

*C_t_* = *ω_a_* × C_a_ + *ω_b_* × C_b_ + *ω_c_* × C_c_ + *ω_d_* × C_d_ + *ω _e_* × C_e_ + *ω_f_* × C_f_ + *ω_g_* × C_g_ (3)

where *j* and *ω_j_*, respectively, represent each analytes and the ratio of AUC_0−∞_ of single analyte to the total analytes; and *C_t_* was self-defined integrated concentration of total analytes after weight factor correction.

## 3. Results and Discussion

### 3.1. Enrichment and Analysis Strategy for Chemical Constituents from the Extract of YPF

The first step of this work was to develop an efficient method for enrichment and analysis of chemical constituents in the extracts of YPF, since the constituents are in wide range of polarity and molecular weight, and often co-eluted with analogs in low concentration, great efforts are needed to solve the encountering difficulties during the process of isolation and detection.

**Figure 1:**
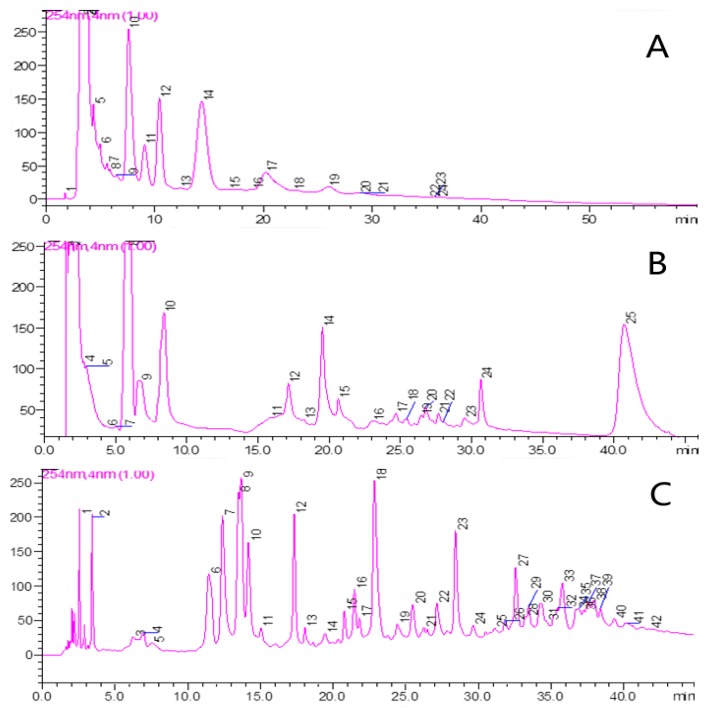
The ^1^D-NPLC chromatograms of YPF decoction on hydrophilic column: (**A**) CN; (**B**) Hilic; and (**C**) Amide.

Firstly, ^2^D-NPLC/RPLC system based on two dimensions with orthogonal separation mechanisms was developed aiming at enhancing the peak capacity of system. Given the complexity of chemical constituents of YPF, we needed to obtain the best peak capacity and maximize the meaningful difference between variable contents of chemicals in two dimensional system. The primary consideration is complete orthogonal separation mechanism, which is currently hard to achieve in reality; the most common used system consists of normal phase chromatographic columns combine with C18 (reverse phase) column. In addition, the major components of YPF decoction were all water-soluble, thus we evaluate the separation efficiency of CN (Figure 1A), Halo Hilic (hydrophilic interaction liquid chromatography) (Figure 1B), and TSKgel Amide (Figure 1C) columns (normal phase chromatographic columns), which are hydrophilic as the first dimension because all separate substances based on differences in hydrophilicity. The ^1^D-NPLC chromatograms of the crude decocted extract of YPF were shown on Figure 1C. Almost 42 peaks could be seen clearly. It is obvious that amide column has the best peak resolution and sensitivity compared with the other two. The packing material of amide column is carbamoyl instead of aminopropyl groups bonded to silica gel, the bonded phase was significantly efficient in analysis of polysaccharides and glycosides, which exist in the herbs *Astragali radix* [12,13], *Atractylodis rhizoma* [17] and *Saposhnikoviae radix* [22], as well as the flavonoids, lactones, volatile oil, etc. [13].

The fractions of YPF water decoction were collected and concentrated after separation in the first NPLC system respectively, which would increase the concentration of the sample. Samples diluted by mobile phase of first dimension would reduce the detection limit of the second dimension. In order to overcome this shortcoming as well as eliminate the influence of solvent incompatibility, this experiment adopted the method of concentrating samples manually, each fraction eluted from amide column in the same period of time were collected after 10 times sample injection, and concentrated to dry under nitrogen, then dissolved in 100 μL methanol–water (1:1, *v*/*v*) for analysis by second dimension chromatography.

**Figure 2:**
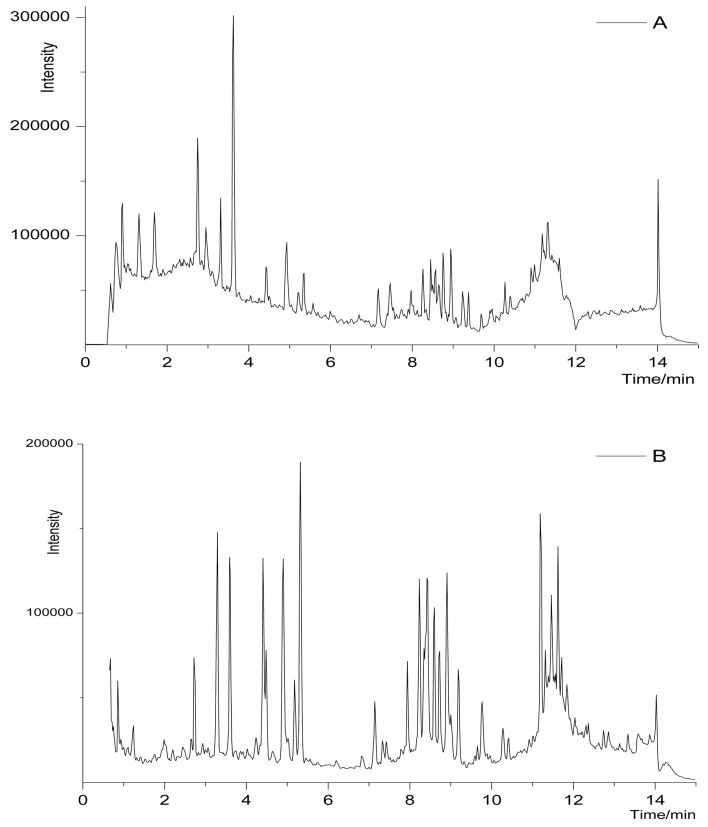
Representative chromatograms of YPF in positive ion mode: (**A**) ^1^D-UPLC-QTOF-MS and chromatograms of second fraction (8–16 min); and (**B**) ^2^D-NPLC/UPLC-QTOF-MS.

To further analyze and characterize each fraction, the high resolution tandem mass spectrometry, particularly QTOF-MS method which can offer great mass accuracy and selectivity with full-scan mode, should be considered. Hence, UPLC-QTOF-MS using C18 chromatographic columns (as the second dimension) was established to isolate the five fractions from ^1^D-NPLC. A representative ^2^D-NPLC/UPLC-QTOF-MS chromatogram (Figure 2B) of second fraction (8–16 min) from ^1^D-NPLC can be seen in Figure 2. For comparison, a ^1^D-UPLC-QTOF-MS chromatogram of the crude decocted extract of YPF is also shown (Figure 2A). It is obvious that the ^2^D method has successfully enriched the peak capacity and improved the resolution as well. There are 3306 molecular features presented in total from all five fractions. ^2^D-NPLC/UPLC-QTOF-MS method was proven to be efficient in analysis of chemical constituents from the extract of YPF.

### 3.2. Characterization of Chemical Constituents of YPF by Using UPLC-QTOF-MS

**Figure 3:**
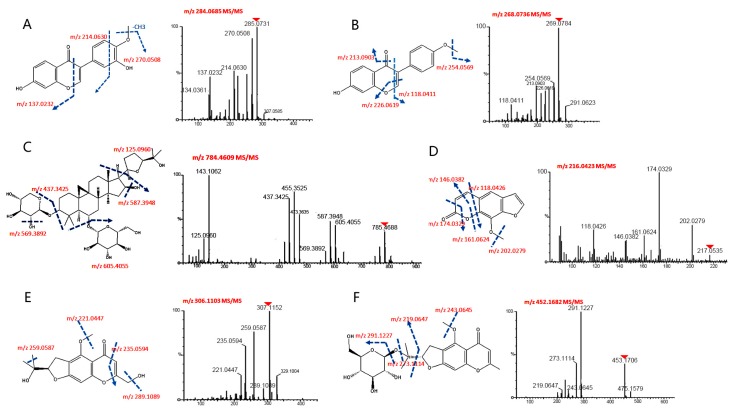
Proposed structures and MS/MS spectra for selected typical compounds. (Parent ions ([M + H]^+^) for MS/MS spectra were marked with red triangles): (**A**) C_16_H_12_O_5_-Calycosin; (**B**) C_16_H_12_O_4_-Fermlononetin; (**C**) C_41_H_68_O_14_-Astragaloside-A; (**D**) C_12_H_8_O_4_-8-Methoxypsoralen; (**E**) C_16_H_18_O_6_-Cimifugin; and (**F**) C_22_H_28_O_10_-4-*O*-beta-d-glucosyl-5-*O*-methylvisamminol.

A large number of chemical constituents were effectively separated from the crude extracts of YPF through the ^2^D-NPLC/UPLC-QTOF-MS system and characterized using UPLC-QTOF-MS, and all of their accurate masses, different retention times and abundances were extracted. However, some compounds were detected overlapped in the five fractions. All compound structures were characterized based on their chromatographic data and multiple fragmentations for structural information, comparing to their standards or referring to previous literature. Some ion mass spectra and the corresponding fragmentation pathway of typical substances are shown in Figure 3. Ultimately, 33 constituents (Table 1, Table 2 and Table 3) were identified or tentatively characterized, including 15 constituents (isoflavones, flavonoids, saponins and glycosides) in *Astragali radix*; seven constituents (atractylodes lactone class, polysaccharide) in *Atractylodis rhizoma*; and 11 constituents (chromone and glycoside) in *Saposhnikoviae radix*.

### 3.3. Quantitative Analysis of Seven Major Active Constituents in YPF in Plasma Samples by UPLC-QqQ-MS

After 33 chemical constituents were identified in the extract of YPF, seven major constituents were chosen to further investigate the pharmacokinetic behavior of YPF formula: astragaloside, calycosin and formononetin in *Astragali radix*; atractylenolide II in *Atractylodes rhizoma*; and cimicifugoside, 4-*O*-beta-d-glucosyl-5-*O*-methylvisamminol, and sec-*O*-glucosylhamaudol in *Saposhnikoviae radix*. In early literature, some pharmacological studies [26,27,28,29,30,31] have proven that these seven constituents are the main active compounds in the corresponding individual herbs, leading to changes of overall therapeutic effects, and they show remarkable anti-inflammatory, antiviral and immunosuppressive activities. Therefore, we assume that these constituents could explain the effectiveness of YPF formula.

However, it is challenging to simultaneously detect these seven compounds in biological samples because of the complicated metabolism in vivo and wide difference in the level of concentration. A proposed UPLC-QqQ-MS could offer much lower limit of detection, making it possible to analyze. Thus, we optimized the most abundant ions and appropriate mass spectrum parameters for analysis after directly injecting a standard solution of each compound using the UPLC-QqQ-MS system. The list of selected SRM parameters, detect mode, cone voltage (CV), collision energy (CE) and retention time (RT) for each analyte is presented at Table 4. Full-scan product ion spectra of Astragaloside (A), Calycosin (B), Formononetin (C), Atractylenolide II (D), Cimicifugoside (E), 4-*O*-beta-d-glucosyl-5-*O*-methylvisamminol (F), and Sec-*O*-glucosylhamaudol (G) are shown in Figure S2.

In order to make a shorter analysis time and good chromatographic behavior and appropriate ionization, we used several volatile additives, such as formic acid, acetic acid, and ammonium acetate, in our experiment. It was finally found that the addition of 0.1% acetic acid to aqueous mobile phase provides a better sensitivity and peak symmetry, and efficiently improves the ionization of all the analytes. Meanwhile, acetonitrile has been proven to provide more stable and practical column back-pressure at the flowrate of 0.5 mL/min than methanol. When considering the initial percentage of organic mobile phase, we found that 5% acetonitrile can significantly change the peak shape of the analytes than other percentage.

Subsequently, sample pre-treatment procedures were also investigated. Protein precipitation by methanol, acetonitrile, ethanol, dimethyl sulfoxide and mixed organic reagent were employed, and methanol and acetonitrile showed satisfied extraction recoveries and matrix effect for all analytes within a minimum number of steps. Better yet, acetonitrile permitted a uniform extraction recoveries and a lower background noise.

### 3.4. Method Validation

The method was fully validated in accordance with the US-FDA document and other related guidelines with respect to specificity, linearity, precision and accuracy, recovery, matrix effect and stability.

**Figure 4:**
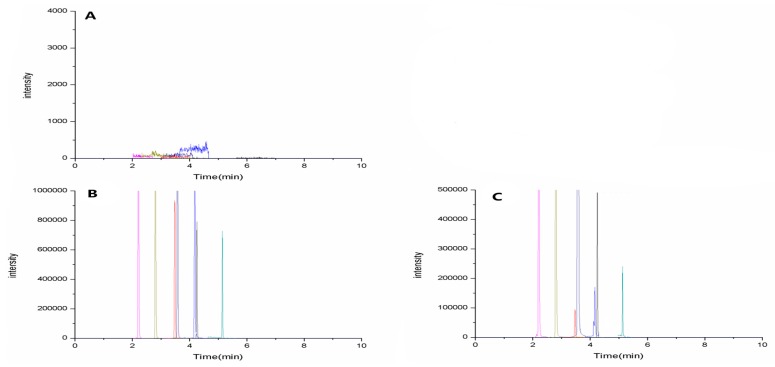
Representative chromatograms of seven analytes, Astragaloside (4.25 min), Calycosin (3.48 min), Formononetin (4.18 min), Atractylenolide II (5.14 min), Cimicifugoside (2.21 min), 4-*O*-beta-d-glucosyl-5-*O*-methylvisamminol (2.80 min), and Sec-*O*-Glucosylhamaudol (3.58 min) in: (**A**) blank plasma; (**B**) blank plasma spiked with the analytes; and (**C**) a plasma sample from a normal rat 15 min after oral administration of mix-std.

Selectivity: Six blank samples of rat plasma from different sources were analyzed. Representative sMRM chromatograms of Astragaloside (4.25 min), Calycosin (3.48 min), Formononetin (4.18 min), Atractylenolide II (5.14 min), Cimicifugoside (2.21 min), 4-*O*-beta-d-glucosyl-5-*O*-methylvisamminol (2.80 min), and Sec-*O*-Glucosylhamaudol (3.58 min) are shown in Figure 4, including blank plasma (Figure 4A), blank plasma spiked with the analytes (Figure 4B), and plasma sample from a normal rat 15 min after oral administration of mix-std (Figure 4C). The background noise was very low, and the absence of endogenous interference at or close to the retention time of the analytes clearly proves the high selectivity of the method.

Linearity and sensitivity: Lowest limit of quantification (LLOQ) is calculated as the lowest level of the analytes that can be reliably detected with a signal-to-noise (S/N) ratio above 10, and reproduced with an accuracy of 80–120%. Summary of regression equations, coefficient and LLOQs of the seven analytes in blank rat plasma are presented in Table 5. All calibration curves exhibited good linearity with coefficient of determination (r^2^) within the range of 0.995–0.999. The LLOQs were sensitive enough for quantitative detection of analytes in the pharmacokinetic studies.

Accuracy and precision: Intra-day and inter-day precision was expressed as the relative standard deviation (RSD) should not exceed 15% (or ±20% for LLOQ), and the accuracy was denoted by the proximity to the theoretical concentration relative error (RE) ((true value of concentration − observed value of concentration)/(true value of concentration) × 100%) should be within ±15% (or ±20% for LLOQ).

Table 6 summarizes the accuracy, precision, recovery and matrix effect data of the seven analytes with different concentrations of low, medium and high in rat plasma (*n* = 5), Intra-day and inter-day precision was less than 13% in terms of RSD%, and the accuracy (RE%) was below 15%, with only one exception of cimicifugoside (17%) at its lowest concentration, which is still be acceptable (≤20%). These values indicate that the overall reproducibility of the method is within the bioanalytical method validation acceptance criteria as per FDA and EMA guidelines.

Matrix effect and recovery: The results of matrix effect and recovery are also summarized in Table 6, from which we can see that the extraction recoveries of analytes and matrix effect are all in the range of 75–120%, with only the recovery of calycosin at 122%, a little bit over the criteria. The other results show this method was consistent, precise and reproducible.

Stability: The analytes would be considered stable when the accuracy bias was within ±15% of the nominal concentrations (or ±20% for LLOQ). Table 7 shows the results of stability studies under different conditions: the measured concentrations for seven analytes at each QC level were within ±13.5% of nominal values, indicating that they were stable under all conditions tested.

### 3.5. Pharmacokinetics in Rats

Because these seven analytes are all difficult to dissolve in water, 0.5% carboxymethyl cellulose sodium was used to make a suspension for oral administration. The dose of 10 mg/kg mix-standard was determined by our previous pharmacokinetic experiments of YPF decoction.

**Figure 5:**
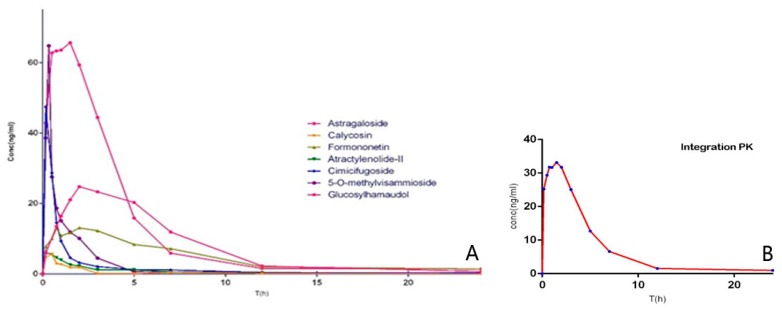
Pharmacokinetic profiles (plasma concentration–time curves) for: seven analytes separately (**A**); and (**B**) their integration.

Mean plasma concentration–time curves of astragaloside, calycosin, formononetin, atractylenolide II, cimicifugoside, 4-*O*-beta-d-glucosyl-5-*O*-methylvisamminol, and sec-*O*-glucosylhamaudol are presented in Figure S3. Moreover, the integrated pharmacokinetics profile is showed in Figure 5, while the results of AUC_0−∞_ and corresponding weighting coefficients (*ωj*) of seven analytes after oral administration and the main pharmacokinetic parameters are listed in Table 8 and Table 9, respectively.

The value of C_max_ and AUC_0-∞_ of cimicifugoside, 4-*O*-beta-d-glucosyl-5-*O*-methylvisamminol and sec-*O*-glucosylhamaudol were found higher than those of other compounds, one of the main reasons is that the glycoside counterparts can be absorbed through intestinal epithelium more efficiently than the aglycones, thus the aglycones show biological activities after being metabolized from their glycoside counterparts in vivo. They were all better fitted to a two-compartment model [32].

In contrast, the relatively low plasma concentrations of atractylenolide II was consistent with previous report, which showed that atracylenolide II has poor intestinal absorption and rapid biliary excretion [33,34]. Because of astragaloside’s low bioavailability characteristics, it could be difficult to detect in rat plasma with such low concentration. With T_max_ at 1.65 h and T_1/2α_ at 1.77 h, formononetin was absorbed and eliminated more slowly than other isoflavones such as calycosin.

Taken together, the results suggests the constituents of *Saposhnikoviae radix* have demonstrated the best absorption and pharmacokinetic behavior among all the major active components in the three medicinal herbs, thus they may play significant role in leading to the main changes of overall therapeutic effects of YPF. However, as discussed above, *Astragali radix* constitutes the most important part of YPF prescription, and we chose only seven main ingredients that have clear anti-inflammatory activity in YPF formula in this study. Therefore, it has its own limitations. We believed that plasma drug concentration of certain points can be regarded as the combination of all quantitative components in vivo, but, considering the huge differences of drug distribution and rate of elimination, the combination will be more complicated than simple multiplication, since the parameter of area under the plasma concentration–time curve from zero to infinity (AUC_0−∞_) is very important for drug efficacy and pharmacology, it can reflects the drug exposure directly. Meanwhile, to measure the intracorporal drug exposure contribution of each component, we choose the ratio of single to overall components′ AUC_0−∞_ as the weighting factor. Ct is self-defined integrated plasma drug concentration of total components after weight factor correction. The integrated pharmacokinetic curve shows that the integrated pharmacokinetic characteristics are different from that of any of the single ingredients, which indicates that the single ingredient pharmacokinetic characteristics cannot fully reflect the drug metabolism of the overall medicine formula in vivo. Further study is needed to confirm the association between them. We hope to provide a new valuable method for the drug metabolism research of traditional Chinese medicine.

## 4. Conclusions

In the present study, we develop a ^2^D-NPLC-UPLC-QTOF-MS platform to separate and identify chemical compounds in YPF. A total of 33 compounds were identified or tentatively characterized, including 15 constituents (isoflavones, flavonoids, saponins and glycosides) from *Astragali radix*; seven constituents (atractylodes lactone class, polysaccharide) from *Atractylodis rhizoma*; and 11 constituents (chromone and glycoside) from *Saposhnikoviae radix*. Then, a rapid, simple and reproducible UPLC-QqQ-MS method was developed for simultaneous quantification of seven major constituents, astragaloside, calycosin, formononetin, atractylenolide II, cimicifugoside, 4-*O*-beta-d-glucosyl-5-*O*-methylvisamminol, and sec-*O*-glucosylhamaudol, in rat plasma with relatively low concentrations. This method was successfully validated and applied to a preclinical pharmacokinetic study. In addition, we also used the area under the plasma concentration–time curve from zero to infinity (AUC_0−∞_) as the weighting factor to make an integrated pharmacokinetic curve, which may offer a broader perspective for discovering and elucidating the pharmacokinetic behavior and clinical application of the whole YPF formula.

## Figures and Tables

**Table 1:** The tentatively identified chemical constituents of *Astragali radix*.

Formula	DBE	Accurate M	[M + H]^+^	Measured [M + H]^+^	Diff (ppm)	Score	Identification
C_16_H_12_O_5_	11	284.0685	285.0763	285.0731	−3.2	96.32	Calycosin
C_22_H_22_O_10_	12	446.1213	447.1291	447.1262	−2.9	97.22	Calycosin-7-glucoside
C_16_H_12_O_4_	11	268.0736	269.0814	269.0784	−3.0	95.43	Fermlononetin
C_22_H_22_O_9_	12	430.1264	431.1342	431.1330	−1.2	98.28	Ononin
C_15_H_10_O_5_	11	270.0528	271.0606	271.0625	1.9	98.99	Baicalein
C_23_H_26_O_10_	11	462.1526	463.1604	463.1620	1.6	97.80	7,2′-dihydroxy-3′,4′-dimethoxyisoflavane-7-*O*-glucoside
C_41_H_68_O_14_	8	784.4609	785.4687	785.4688	0.1	99.92	Astragaloside A
C_45_H_72_O_16_	10	868.4820	869.4899	869.4891	−0.8	99.03	Isoastragaloside I
C_43_H_70_O_15_	9	826.4715	827.4793	827.4752	−4.1	93.65	Isoastragaloside II
C_15_H_14_O_9_	9	338.0638	339.0716	339.0732	1.6	97.98	Quercetin dihydrate
C_16_H_12_O_7_	11	316.0583	317.0661	317.0699	3.8	95.64	Isorhamnetin
C_16_H_12_O_6_	11	300.0634	301.0712	301.0752	4.0	92.37	Rhamnocitrin
C_10_H_7_ClN_2_O_2_S	7	253.9917	254.9995	254.9981	−1.4	99.01	Astragalus Polysaccharide
C_30_H_50_O	6	426.3862	427.3940	427.3894	−4.6	95.34	Lupeol
C_15_H_10_O_5_	11	270.0528	271.0606	271.0636	3.0	96.78	Apigenin

**Table 2:** The tentatively identified chemical constituents of *Atractylodes rhizoma*.

Formula	DBE	Accurate M	[M + H]^+^	Measured [M + H]^+^	Diff (ppm)	Score	Identification
C_6_H_14_O_6_	0	182.0790	183.0869	183.0888	1.9	98.44	d-Mannitol
C_15_H_20_O_2_	6	232.1463	233.1542	233.1513	−2.9	97.73	Atractylenolide II
C_18_H_26_O_3_	6	290.1882	291.1960	291.1899	−6.1	92.35	8β-Ethoxy Atractylenolide III
C_15_H_20_O	6	216.1514	217.1592	217.1608	1.6	98..29	Atractyline
C_13_H_10_O	9	182.0732	183.0810	183.0837	2.7	97.40	Atractylodin
C_32_H_52_O_2_	7	468.3967	469.4046	469.4013	−3.3	96.56	Erythrodiol 3-acetate
C_29_H_50_O	5	414.3862	415.3940	415.3901	3.9	96.14	Beta-Sitosterol

**Table 3:** The tentatively identified chemical constituents of *Saposhnikoviae radix*.

Formula	DBE	Accurate M	[M + H]^+^	Measured [M + H]^+^	Diff (ppm)	Score	Identification
C_12_H_8_O_4_	9	216.0423	217.0501	217.0535	3.4	95.88	8-Methoxypsoralen
C_11_H_6_O_3_	9	186.0317	187.0395	187.0389	0.6	99.39	Psoralen
C_11_H_10_O_5_	7	222.0528	223.0606	223.0624	1.8	98.05	Isofraxidin
C_10_H_8_O_4_	7	192.0423	193.0501	193.0541	4.0	93.99	Scopoletin
C_16_H_14_O_4_	10	270.0892	271.0970	271.0957	1.3	99.21	Imperatorin
C_17_H_18_O_6_	9	318.1103	319.1182	319.1201	1.9	98.10	3-*O*-Acetylhamaudol
C_20_H_22_O_6_	10	358.1416	359.1495	359.1510	1.5	98.06	3-*O*-Angeloylhamaudol
C_16_H_18_O_6_	8	306.1103	307.1182	307.1152	−3.0	96.77	Cimifugin
C_22_H_28_O_11_	9	468.1632	469.1710	469.1718	0.8	98.71	Cimicifugosid
C_21_H_26_O_10_	9	438.1526	439.1604	439.1598	0.6	98.86	Sec-*O*-glucosylhamaudol
C_22_H_28_O_10_	9	452.1682	453.1761	453.1706	−5.5	92.99	4-*O*-beta-d-glucosyl-5-*O*-methylvisamminol

Products identified by comparing with reference standards and fragmentation pathway are in bold.

**Table 4:** List of selected SRM parameters, detect mode, cone voltage (CV), collision energy (CE) and retention time (RT) for each analyte

Analyte	Q1 Mass (Da)	Q3 Mass (Da)	Detect Mode	DP (v)	CE (eV)	RT (min)
Astragaloside	785.5	143.0	ESI+	22	17	4.19
Calycosin	285.2	270.1	ESI+	76	31	3.50
Formononetin	269.2	213.1	ESI+	70	32	4.15
Atractylenolide II	233.2	187.2	ESI+	57	23	5.13
Cimicifugoside	469.2	307.3	ESI+	66	39	2.21
4-*O*-beta-d-glucosyl-5-*O*-methylvisamminol	453.2	291.3	ESI+	77	30	2.81
Sec-*O*-Glucosylhamaudol	439.2	277.2	ESI+	71	21	3.58

**Table 5:** Summary of regression equations, coefficient and LLOQs of the seven analytes in rat plasma.

Analyte	Regression Equation	r^2^	LLOQ (ng/mL)
Astragaloside	*Y* = 36184*x* + 1385	0.999	0.06
Calycosin	*Y* = 44363*x* + 5319	0.999	0.06
Formononetin	*Y* = 5346*x* – 2304	0.997	0.09
Atractylenolide II	*Y* = 119887*x* + 7926	0.999	0.03
Cimicifugoside	*Y* = 114555*x* + 124528	0.995	0.01
4-*O*-beta-d-glucosyl-5-*O*-methylvisamminol	*Y* = 210524*x* + 106757	0.997	0.01
Sec-*O*-Glucosylhamaudol	*Y* = 326228*x* + 92710	0.998	0.01

**Table 6:** Summary of accuracy, precision, recovery and matrix effect of the seven analytes in rat plasma (*n* = 5).

Analyte	Concentration (ng/mL)	Intra-Day RSD (%)	Inter-Day RSD (%)	Accuracy (RE %)	Recovery (%, mean)	Matrix Effect (%, Mean)
Astragaloside	0.25	7.06	12.69	3.26	83.91	109.25
	3.50	1.06	5.12	−8.84	106.36	74.47
	150	1.45	4.30	0.63	94.54	100.88
Calycosin	0.25	4.18	8.27	1.45	104.90	69.72
	3.50	2.20	3.82	3.12	113.35	62.99
	150	4.38	9.59	−5.38	122.48	109.58
Formononetin	1.50	2.35	4.91	9.82	95.30	114.39
	15.0	2.33	4.98	2.31	100.03	98.63
	80.0	2.82	5.87	-----	96.09	105.72
Atractylenolide II	0.50	3.87	8.76	15.30	82.25	105.48
	1.50	2.17	5.43	0.32	85.28	106.33
	80.0	2.94	7.25	−1.99	83.47	103.27
Cimicifugoside	0.25	2.78	5.60	17.31	99.12	112.22
	3.50	2.99	7.01	10.50	106.27	92.79
	150	1.07	3.36	3.43	97.63	99.46
4′-*O*-beta-d-glucosyl-5-*O*-methylvisamminol	0.50	3.89	8.13	6.84	87.94	99.91
	1.50	1.35	3.28	5.23	98.10	97.73
	80.0	1.02	3.71	3.95	95.83	105.89
Sec-*O*-Glucosylhamaudol	0.50	2.75	5.11	−12.51	92.97	96.82
	1.50	1.40	3.99	7.06	98.70	84.76
	80.0	1.01	2.48	5.59	96.86	101.23

**Table 7:** Summary of Stability of the seven analytes in rat plasma (*n* = 6).

Analyte	Concentration (ng/mL)	Short-Term RSD (%)	Long-Term RSD (%)	Freeze-Thaw RSD (%)
Astragaloside	0.25	7.06	3.36	3.65
	3.50	1.06	8.08	3.20
	150	1.45	2.80	11.51
Calycosin	0.25	4.18	4.13	8.29
	3.50	2.20	10.35	6.08
	150	4.38	12.67	12.92
Formononetin	1.50	2.35	5.96	10.06
	15.0	2.33	7.75	7.09
	80.0	2.82	6.22	10.58
Atractylenolide II	0.50	3.87	4.11	7.18
	1.50	2.17	9.76	5.77
	80.0	2.94	3.37	13.55
Cimicifugoside	0.25	2.78	2.80	3.97
	3.50	2.99	4.11	1.36
	150	1.07	1.59	8.77
4-*O*-beta-d-glucosyl-5-*O*-methylvisamminol	0.50	3.89	3.99	2.40
	1.50	1.35	7.52	1.40
	80.0	1.02	2.41	8.53
Sec-*O*-Glucosylhamaudol	0.50	2.75	3.70	4.77
	1.50	1.40	3.11	3.73
	80.0	1.01	1.46	9.64

**Table 8:** AUC_0−∞_ and corresponding weighting coefficients (*ω_j_*) of seven analytes after oral administration (10 mg/kg).

Parameters	A	B	C	D	E	F	G
AUC_0−∞_ (ng·h/mL)	191.0	11.5	113.0	17.9	45.8	60.8	269.3
*ω_j_*	0.269	0.016	0.159	0.025	0.064	0.085	0.379

**Table 9:** Pharmacokinetic parameters of analytes and integrated PK after oral administration (10 mg/kg) to rats (*n* = 6, Mean ± S.D.).

Analyte	C_max_ (ng/mL)	T_max_ (h)	AUC_0−∞_ (ng·h/mL)	CL/F (L/h·kg)	T_1/2α_ (h)	T_1/2β_ (h)
Astragaloside	26.6 ± 4.0	2.8 ± 0.1	191.0 ± 45.1	49.9 ± 5.8	1.98 ± 0.11	2.06 ± 0.04
Calycosin	6.3 ± 2.5	0.3 ± 0.1	11.5 ± 6.7	499.9 ± 135.0	0.27 ± 0.10	1.09 ± 0.36
Formononetin	13.9 ± 4.2	1.6 ± 0.9	113.0 ± 23.9	91.9 ± 19.9	1.77 ± 0.81	4.10 ± 2.60
Atractylenolide II	8.4 ± 2.4	0.1 ± 0.1	17.9 ± 4.9	589.0 ± 142.0	0.04 ± 0.01	4.10 ± 1.56
Cimicifugoside	48.7 ± 8.4	0.2 ± 0.01	45.8 ± 7.5	223.5 ± 38.6	0.10 ± 0.04	3.80 ± 1.57
4-*O*-beta-d-glucosyl-5-*O*-methylvisamminol	50.0 ± 7.6	0.2 ± 0.01	60.8 ± 9.9	168.4 ± 30.9	0.16 ± 0.01	2.40 ± 1.10
Sec-*O*-Glucosylhamaudol	67.1 ± 1.7	1.1 ± 0.2	269.3 ± 68.2	39.1 ± 9.6	1.10 ± 0.26	0.04 ± 0.02
Integration PK	32.83	1.64	375.30	26.60	1.67	155.30

## Supplementary Materials

The following are available online. Figure S1: Medicinal plants of YPF. Figure S2: Full-scan product ion spectra of: (**A**) Astragaloside; (**B**) Calycosin; (**C**) Formononetin; (**D**) Atractylenolide II; (**E**) Cimicifugoside; (**F**) 4-*O*-beta-d-glucosyl-5-*O*-methylvisamminol; and (**G**) Sec-*O*-Glucosylhamaudol. Figure S3: Mean plasma concentration–time curves for: (**A**) Astragaloside; (**B**) Calycosin; (**C**) Formononetin; (**D**) Atractylenolide II; (**E**) Cimicifugoside; (**F**) 4-*O*-beta-d-glucosyl-5-*O*-methylvisamminol; and (**G**) Sec-*O*-Glucosylhamaudol from a normal rat after oral administration of mix-std.

## References

[1] Hu C., Xu G. (2014). Metabolomics and traditional Chinese medicine. TrAC Trends Anal. Chem..

[2] Xiao S., Luo K., Wen X., Fan X., Cheng Y. (2014). A pre-classification strategy for identification of compounds in traditional Chinese medicine analogous formulas by high-performance liquid chromatography-mass spectrometry. J. Pharm. Biomed. Anal..

[3] Ye L., Jia Y., Ji K.E., Sanders A.J., Xue K., Ji J., Mason M.D., Jiang W.G. (2015). Traditional Chinese medicine in the prevention and treatment of cancer and cancer metastasis. Oncol. Lett..

[4] Zhong Y., Menon M.C., Deng Y., Chen Y., He J.C. (2015). Recent Advances in Traditional Chinese Medicine for Kidney Disease. Am. J. Kidney Dis..

[5] Zhang A., Sun H., Wang X. (2014). Potentiating therapeutic effects by enhancing synergism based on active constituents from traditional medicine. Phytother. Res..

[6] Zhang A., Sun H., Qiu S., Wang X. (2013). Advancing drug discovery and development from active constituents of yinchenhao tang, a famous traditional Chinese medicine formula. Evid. Based Complement. Altern. Med..

[7] Hao H., Zheng X., Wang G. (2014). Insights into drug discovery from natural medicines using reverse pharmacokinetics. Trends Pharmacol. Sci..

[8] Song J., Li J., Zheng S.R., Jin Y., Huang Y. (2013). Anti-inflammatory and immunoregulatory effects of Yupingfeng powder on chronic bronchitis rats. Chin. J. Integr. Med..

[9] Liu Q., Lu L., Hua M., Xu Y., Xiong H., Hou W., Yang Z. (2013). Jiawei-Yupingfeng-Tang, a Chinese herbal formula, inhibits respiratory viral infections in vitro and in vivo. J. Ethnopharmacol..

[10] Huang J.H., Mu Z.L., Zhou X.J., Huang Q.L., Gao F., Chen X. (2015). Effect of Yupingfeng granules on HA and Foxp3(+) Treg expression in patients with nasopharyngeal carcinoma. Asian Pac. J. Trop. Med..

[11] Zhao P., Su G.H., Xiao X.Y., Hao E.K., Zhu X.L., Ren J. (2008). Chinese medicinal herb *Radix astragali* suppresses cardiac contractile dysfunction and inflammation in a rat model of autoimmune myocarditis. Toxicol. Lett..

[12] Guo S.B., Du X.M., Jian L.Y. (2014). Studies on purification process of total Saponins in *Radix astragali* with resin and structural identification of compounds. Asian J. Chem..

[13] Chu C., Qi L.W., Liu E.H., Li B., Gao W., Li P. (2010). *Radix astragali* (*Astragalus*): Latest advancements and trends in chemistry, analysis, pharmacology and pharmacokinetics. Curr. Org. Chem..

[14] Fu X., Song B., Tian G.W., Li J.L. (2014). The effects of the water-extraction of *Astragali radix* and Lycopi herba on the pathway of TGF-smads-UPP in a rat model of diabetic nephropathy. Pharmacogn. Mag..

[15] Zhou R.N., Song Y.L., Ruan J.Q., Wang Y.T., Yan R. (2012). Pharmacokinetic evidence on the contribution of intestinal bacterial conversion to beneficial effects of Astragaloside IV, a marker compound of *Astragali radix*, in traditional oral use of the herb. Drug Metab. Pharmacokinet..

[16] Chin Y.-W., Jung Y.H., Chae H.-S., Yoon K.D., Kim J. (2011). Anti-inflammatory constituents from the roots of *Saposhnikovia divaricata*. Bull. Korean Chem. Soc..

[17] Meng H., Li G.Y., Dai R.H., Ma Y.P., Zhang K., Zhang C., Li X.A., Wang J.H. (2010). Chemical constituents of *Atractylodes chinensis* (DC.) Koidz. Biochem. Syst. Ecol..

[18] Choi K.H., Il Jeong S., Lee J.H., Hwang B.S., Kim S.J., Lee S., Choi B.K., Jung K.Y. (2011). Pharmacological mechanism responsible for the Atractylodes japonica-induced distal colonic contraction in rats. Phytomedicine.

[19] Li C.Q., He L.C., Dong H.Y., Jin J.Q. (2007). Screening for the anti-inflammatory activity of fractions and compounds from *Atractylodes macrocephala* koidz. J. Ethnopharmacol..

[20] Wang H.Z., Wang L., Zhang N., Zhang Q., Zhao H., Zhang Q.X. (2014). Houshiheisan compound prescription protects neurovascular units after cerebral ischemia. Neural Regen. Res..

[21] Chen Q.H., Li P., Li B., He H.S., Yang H.D., Zhu J. (2013). Characterization and quantification of Prim-*O*-Glucosylcimifugin in the roots of *Saposhnikovia divaricata* and its medicinal preparations by liquid chromatography-ion trap mass spectrometry. J. Liq. Chromatogr. Relat. Technol..

[22] Li L., Gui Y.G., Wang J., Zong X.F., Zhang H.R., Liu C.M. (2013). Preparative separation of chromones in plant extract of *Saposhnikovia divaricata* by reverse-phase medium-pressure liquid chromatography and high performance counter-current chromatography. J. Liq. Chromatogr. Relat. Technol..

[23] Li T., Wang Y., Wang Y., Liang R., Zhang D., Zhang H., Chen L., Yang W. (2013). Development of an SPE–HPLC–MS method for simultaneous determination and pharmacokinetic study of bioactive constituents of Yu Ping Feng San in rat plasma after oral administration. J. Ethnopharmacol..

[24] Chen Q., He H., Li P., Zhu J., Xiong M. (2013). Identification and quantification of atractylenolide I and atractylenolide III in *Rhizoma atractylodes* Macrocephala by liquid chromatographyion trap mass spectrometry. Biomed. Chromatogr..

[25] Ge J., Wang Y.-W., Lu X.-C., Sun X.-H., Gong F.-J. (2007). Determination of atractylenolide II in rat plasma by reversed-phase high-performance liquid chromatography. Biomed. Chromatogr..

[26] Yan H., Sun Y., Zhang Q., Yang M., Wang X., Wang Y., Yu Z., Zhao Y. (2015). Simultaneous determination and pharmacokinetic study of Atractylenolide I, II and III in rat plasma after intragastric administration of Baizhufuling extract and Atractylodis extract by UPLC-MS/MS. J. Chromatogr. B Anal. Technol. Biomed. Life Sci..

[27] Shahzad M., Shabbir A., Wojcikowski K., Wohlmuth H., Gobe G.C. (2016). The antioxidant effects of radix astragali (*Astragalus membranaceus* and Related Species) in protecting tissues from injury and disease. Curr. Drug Targets.

[28] Kai Z., Michela P., Antonio P., Annamaria P. (2015). Biological active ingredients of traditional Chinese herb *Astragalus membranaceus* on treatment of diabetes: A systematic review. Mini-Rev. Med. Chem..

[29] Cheng Y., Mai J.-Y., Hou T.-L., Ping J., Chen J.-J. (2016). Antiviral activities of atractylon from *Atractylodis rhizoma*. Mol. Med. Rep..

[30] Ji G., Chen R., Zheng J. (2014). Atractylenolide I inhibits lipopolysaccharide-induced inflammatory responses via mitogen-activated protein kinase pathways in RAW264.7 cells. Immunopharmacol. Immunotoxicol..

[31] Li Y., Zhao L., Zhang H., Jia J., Lv L., Zhou G., Chai Y., Zhang G. (2012). Comparative pharmacokinetics of prim-*O*-glucosylcimifugin and cimifugin by liquid chromatography-mass spectrometry after oral administration of *Saposhnikoviae radix* extract, cimifugin monomer solution and prim-*O*-glucosylcimifugin monomer solution to rats. Biomed. Chromatogr..

[32] Liu X.H., Zhao J.B., Guo L., Yang Y.L., Hu F., Zhu R.J., Feng S.L. (2014). Simultaneous determination of calycosin-7-*O*-beta-d-glucoside, ononin, calycosin, formononetin, astragaloside IV, and astragaloside II in rat plasma after oral administration of *Radix astragali* extraction for their pharmacokinetic studies by ultra-pressure liquid chromatography with tandem mass spectrometry. Cell. Biochem. Biophys..

[33] Li Y., Zhang Y., Wang Z., Zhu J., Tian Y., Chen B. (2012). Quantitative analysis of atractylenolide I in rat plasma by LC-MS/MS method and its application to pharmacokinetic study. J. Pharm Biomed. Anal..

[34] Shi Y.Y., Guan S.H., Tang R.N., Tao S.J., Guo D.A. (2012). Simultaneous determination of atractylenolide II and atractylenolide III by liquid chromatography-tandem mass spectrometry in rat plasma and its application in a pharmacokinetic study after oral administration of Atractylodes Macrocephala Rhizoma extract. Biomed. Chromatogr..

