# Supplementary material for: Using UPLC-MS/MS for Characterization of Active Components in Extracts of Yupingfeng and Application to a Comparative Pharmacokinetic Study in Rat Plasma after Oral Administration
Source: Molecules. 2017 May 17;22(5):810. doi: 10.3390/molecules22050810 (PMC6154636; doi:10.3390/molecules22050810)
Supplement: Supplementary file 1 [file molecules-22-00810-s001.pdf]

Supplementary Materials: The following are available online at www.mdpi.com/link,

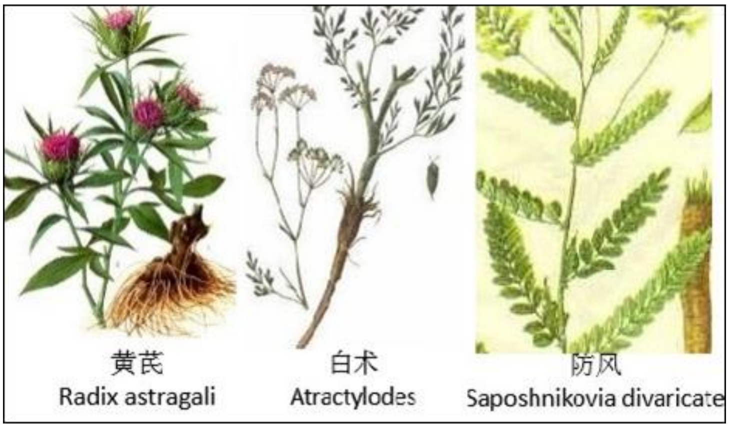

Figure S1. Medicinal plants of YPF.

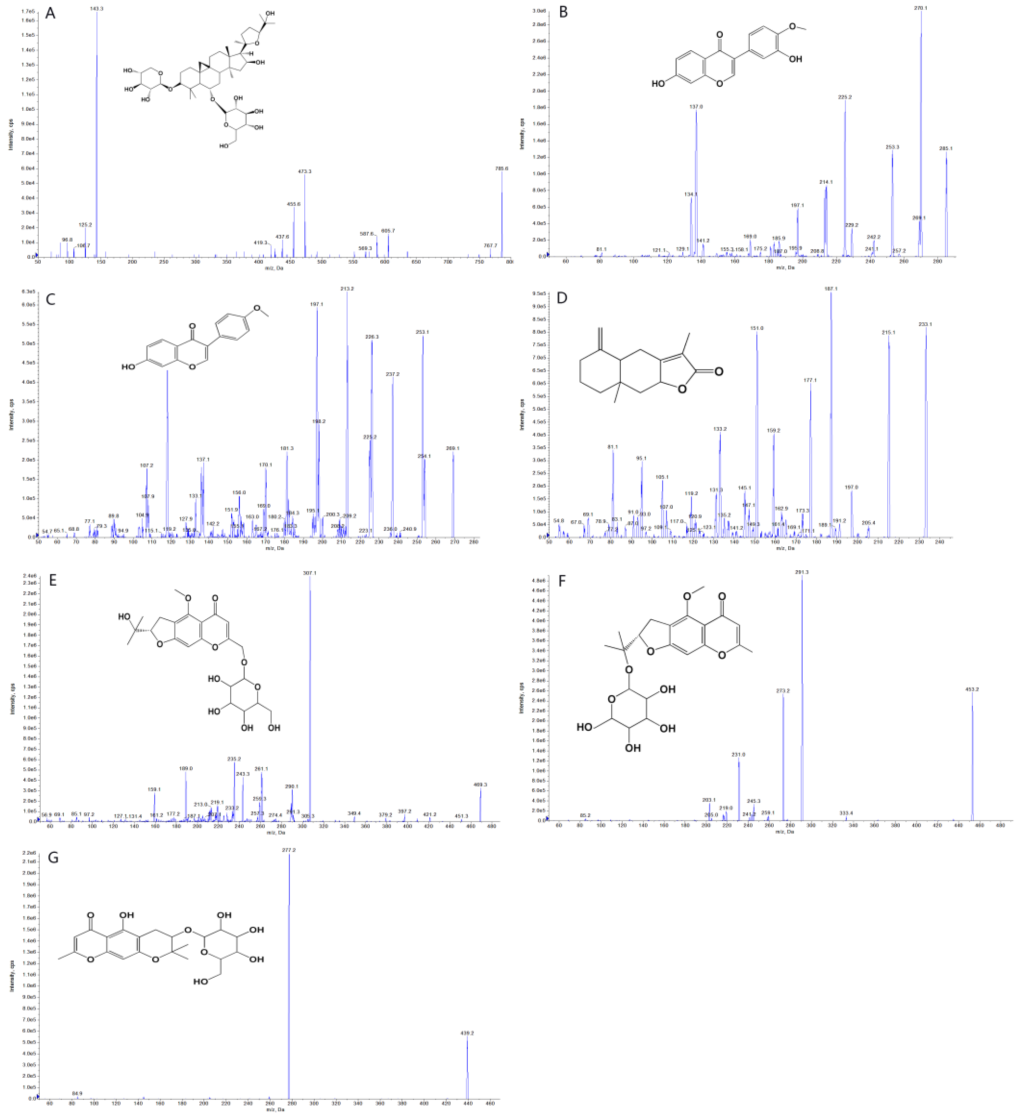

Figure S2. Full-scan product ion spectra of: (**A**) Astragaloside; (**B**) Calycosin; (**C**) Formononetin; (**D**) Atractylenolide II; (**E**) Cimicifugoside; (**F**) 4-O-beta-D-glucosyl-5-O-methylvisamminol; and (**G**) Sec-O-Glucosylhamaudol

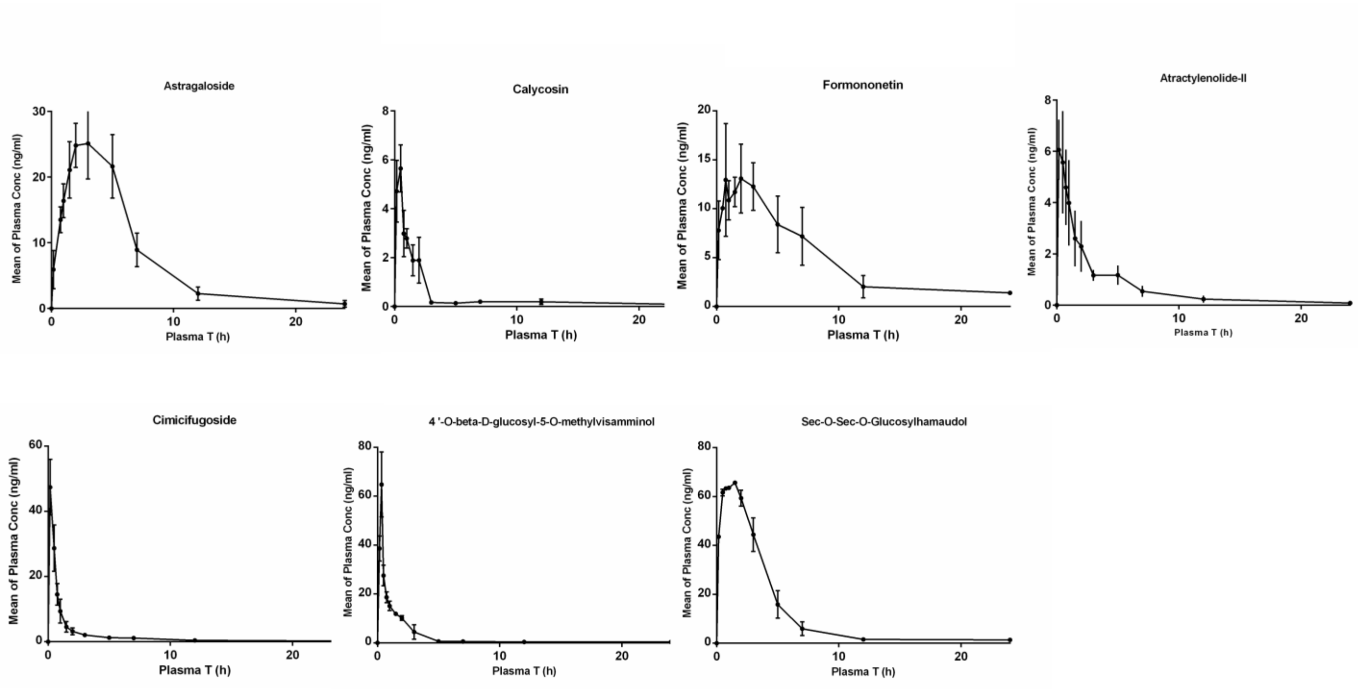

Figure S3. Mean plasma concentration–time curves for: (**A**) Astragaloside; (**B**) Calycosin; (**C**) Formononetin; (**D**) Atractylenolide II; (**E**) Cimicifugoside; (**F**) 4-*O*-beta-D-glucosyl-5-*O*-methylvisamminol; and (**G**) Sec-*O*-Glucosylhamaudol from a normal rat after oral administration of mix-std
